# Distribution of relaxation times as a tool to monitor tissue electroporation

**DOI:** 10.1038/s41598-025-25647-4

**Published:** 2025-11-24

**Authors:** Théo Le Berre, Damien Voyer, Guilhem Rival, Marie Frénéa-Robin, Julien Marchalot

**Affiliations:** 1 Ampere, UMR5005, Ecole Centrale de Lyon, INSA Lyon, CNRS, Universite Claude Bernard Lyon 1, 69130 Ecully, France; 2 EIGSI Lab, 17041 La Rochelle, France; 3 AIMOKA Project, Inria Research Centre, Rennes Universite, 35042 Rennes, France; 4 LGEF, UR682, INSA Lyon, 69621 Villeurbanne, France

**Keywords:** Electroporation, Impedance spectroscopy, Cell membrane, Biophysics, Engineering

## Abstract

In this paper, the effect of electroporation on tissue is studied using the concept of distribution of relaxation times (DRT). DRT is an alternative to traditional electrical impedance spectroscopy, where measurement data are analyzed on a time scale rather than in the frequency domain. Applied to biological tissue, this approach is shown to remove the contribution of the electrode polarization in a 2-electrode system in a simple way. DRT also makes it easy to differentiate between dispersions associated with the different compartments of the tissue (counterion cloud, cell membrane, cell content and cell nucleus) and quantify their contribution to the total DC resistance. The study of plant tissue samples reveals the wide spread of the $\beta$ dispersion related to cell membrane polarization, which can be explained not only by the variable size of cells but also by the variable content of cells. The effects of electroporation can also be analyzed precisely. In particular, the irreversible electroporation threshold varies according to the cell content, as confirmed by electroporation simulations. In the electric field range of reversible electroporation, the shape of the DRT related to the $\beta$ dispersion is significantly modified, while the DC resistance remains constant. One explanation is put forward, involving the counterions cloud whose spatial distribution is altered after the application of the electric field.

## Introduction

Electroporation or electropermeabilisation is a phenomenon whereby the structure of the cell membrane is weakened under the action of an intense electric field. This property is now exploited in therapeutics such as electrochemotherapy, where bleomycin or cisplatin are transported into cells once the membrane has become permeable^1^, or in irreversible electroporation ablation, where cancer cells die notably through apoptosis^2,3^.

Although the efficacy of electroporation therapy is increasing, this technique requires real-time monitoring tools to quantify the effects of the electric field and manage the delivery of high-voltage pulses applied^4^. One of the techniques being considered is electrical impedance spectroscopy (EIS)^5^. This technique has been used for several decades to characterize the properties of biological tissues^6^. It involves measuring impedance over a wide range of frequencies by injecting a low-amplitude signal between electrodes. This impedance reflects electrical conductivity and permittivity, revealing dispersions that characterize phenomena induced at different scales in biological tissue^7^.

One of these phenomena is the interfacial polarization of the cell membrane, associated with the $\beta$ dispersion, which generally appears between 10 kHz and 10 MHz. EIS performed in this frequency range is attractive in therapies using electroporation because electroporation is specifically aimed at modifying the physiological state of membranes in a reversible or irreversible way^8^.

However, biological tissues are complex systems with a high degree of heterogeneity. Cells vary in size and shape; cell content can also vary considerably, as can the extracellular environment. Under these conditions, interfacial polarization is related to a distribution of characteristic times rather than a single one^9^. Overlap of the $\beta$ dispersion with other dispersions is also possible, particularly with the $\alpha$ dispersion, which is mainly due to the response of the counterions at the membrane surface. These tissue characteristics can make it difficult, if not impossible, to extract the response of membrane cells from spectral measurements.

These limitations of EIS have already been pointed out for other complex systems, notably batteries and fuel cells^10^. In those systems, EIS is used to analyze various electrochemical phenomena, such as diffusion or charge transfer, induced at different spatial/time scales. In order to improve battery and fuel cell diagnostic, the concept of distribution of relaxation times (DRT) was introduced with success: it transforms the spectral data obtained by EIS into a distribution of relaxation times related to first-order processes, where it is easier to decouple the different kinetics^11^. To our knowledge, there are still very few applications of DRT to biological tissues. The first case study was proposed in^12^, where DRT is deduced from impedance measurements made with a 4-electrode device; it is used to describe the dispersion of healthy tissues such as liver or lung and a comparison with the Cole Cole model is made to show the relevance of this approach. Very recently, DRT has been combined with a machine learning algorithm to diagnose lung cancer^13^.

In this paper, we propose to analyze the DRT built from impedance data carried out on untreated and electropored potato samples. Compared to a frequency analysis of EIS data, the use of DRT presents a number of advantages when applied to the electroporation of biological tissue, as shown in this paper. The article is organized as follows: the first section briefly describes the theory of DRT; the case where the DRT takes on a complex shape is described more precisely by introducing decomposition with a sum of Gaussian distributions. Material and methods are presented in the second section. In the third section, preliminary impedance measurements are reported with 2- and 4-electrode systems. It is shown that the part of the DRT due to electrode polarization can be identified and simply removed in the case of a 2-electrode system. The fourth section is dedicated to the analysis of measurements on potato samples. Firstly, the DRT of the native potato samples is decomposed into a reduced number of Gaussian distributions, each related to a dispersion in one of the tissue compartments. Secondly, the evolution of the Gaussians after electroporation is monitored as a function of the electric field; the results are interpreted for both reversible and irreversible electroporation.

## Theoretical background

DRT consists in analyzing an impedance spectrum $Z(\omega )$ in terms of a distribution $g(\tau )$ of times $\tau$ related to first order relaxation processes:

1 $$\begin{aligned} Z\left( \omega \right) = R_{\infty } + R_p \int _0^\infty \frac{g(\tau )}{1+j\omega \tau } d\tau \end{aligned}$$

where $\int _0^\infty g(\tau ) d\tau = 1$, $R_{\infty }$ the resistance when $\omega \rightarrow +\infty$ and $R_p$ the polarization resistance that is added when $\omega = 0$, i.e. when the steady state is achieved.

DRT is a mathematical transformation designed to perform data analysis on a time scale. The function $g(\tau )$ is not fixed a priori. This is an important difference from frequency domain approaches. Tissue response is very often described in the frequency domain with a Cole Cole model^14^; other empirical models can be used, notably the model proposed by Havriliak and Negami, which is a generalized form of Cole-Cole dispersion to describe asymmetric dispersions^15^. All these models fix a priori the type of the distribution $g(\tau )$^16^.

Numerically, the unknown distribution $g(\tau )$ is expanded into *M* step functions over a bounded domain $[\tau _{inf},\tau _{sup}]$, divided into constant intervals according to a logarithm scale $log(\tau )$^17^. The weight of the step functions is computed using the set of *N* impedances $Z(\omega )$ measured by spectroscopy. The discretization of the problem leads to a set of *N* linear equations with *M* unknowns. *M* can be larger than *N*, so the problem is generally ill-conditioned. One of the most common solutions is to solve the regression problem by adding a penalty term in order to make the problem well-posed; this penalty term is weighted by a $\lambda$ coefficient that is set according to the quality of the data^11^. In our case, we have forced the solution $g(\tau )$ to be smooth by adding a penalty term $\Vert g'(\tau)\Vert ^2$.

As a logarithm scale is used in the discretization of the relaxation times domain, it is more appropriate to analyze the solution introducing the function $G(log(\tau ))=\tau g(\tau )$ and the new variable $x = log(\tau )$. One has:

2 $$\begin{aligned} \int _{-\infty }^{+\infty } G(x) dx =\int _0^\infty \tau g(\tau ) dlog(\tau ) =\int _0^\infty g(\tau ) d\tau = 1 \end{aligned}$$

When the DRT takes a complex shape, the distribution *G* can be expanded as a sum of Gaussian distributions with respect to the variable $log(\tau )$, or equivalently log-normal distributions with respect to $\tau$. A log-normal distribution can be written as:

3 $$\begin{aligned} p_{logN}(\tau ) =\frac{a}{\tau } exp\left( -\frac{(log(\tau /\mu ))^2}{2\sigma ^2}\right) \end{aligned}$$

where $\mu$ is the mean value of $\tau$, $\sigma ^2$ the variance in the logarithmic scale and *a* the peak value obtained for $\tau = \mu$.

Modeling dispersion using a log-normal distribution in DRT is an alternative to modeling dispersion with a RQ element in impedance spectroscopy. RQ element connects a resistance *R* in parallel with a constant phase element $1/(Q (j \omega )^q)$, with $q<1$. The RQ impedance is $R/(1+R Q (j \omega )^q)$: its frequency response is that of the Cole-Cole model. Interestingly, the DRT of the Cole-Cole model is analytical: it is bell-shaped according to a logarithmic scale of $\tau$, as for a log-normal distribution; however, the Cole-Cole distribution is less spread out than a log-normal distribution^16^.

Assuming that *G* is modeled with *K* Gaussian distributions, the impedance can be expanded as follows:

4 $$\begin{aligned} Z\left( \omega \right) = R_{\infty } + R_p \sum _{k=1}^K \int _0^\infty \frac{p_{logNk}(\tau )}{1+j\omega \tau } d\tau \end{aligned}$$

In addition, one has:

5 $$\begin{aligned} \int _{0}^{\infty } p_{logNk}(\tau ) d\tau =\int _{-\infty }^{+\infty } a_k exp\left( -\frac{(x-log(\mu _k))^2}{2\sigma _k^2}\right) dx = a_k \sigma _k \sqrt{2\pi } \end{aligned}$$

By setting $\omega \rightarrow 0$ in equation (4), the DC resistance can be expanded as follows:

6 $$\begin{aligned} Z\left( 0 \right) = R_0 = R_{\infty } + \sum _{k=1}^K R_{pk} \end{aligned}$$

with $$R_{pk} = R_p a_k \sigma _k \sqrt{2\pi }$$.

$R_{pk}$ values in equation (6) gives the quantitative contribution of the different Gaussians to the DC resistance.

This equation can also be interpreted physically. Each Gaussian is associated with the relaxation related to a tissue compartment (cytoplasm, nucleus, starch granule, etc.). Assume that the Gaussians are ordered so that $\mu _1> \mu _2... > \mu _K$. At infinite frequency, the current density flows through all compartments, leading to the minimum resistance $R_\infty$. As frequency decreases, steady state is reached for more and more relaxation processes: first for process *K*, then process $K-1$, and so on up to process numbered 1. The current then flows through less and less tissue compartments, gradually increasing the resistance by $R_{pK}$, $R_{pK}+R_{p(K-1)}$ and so on.

## Materials and methods

The experiments were carried out using potatoes of the Agata variety harvested in France. They were purchased from a major distributor in March. All measured samples were cut from the core of two potatoes. Between measurements, the not yet used samples were wrapped in damp paper to avoid drying.

### Preliminary measurements—needle electrodes

For the preliminary experiment reported in the next section , the samples were cut from potato slices (approximately 5 mm thick) by hand with a scalpel and placed on a linear array of 2 or 4 stainless steel needles, the size of the sample being significantly larger than the array to avoid any edge effects. The distance between each electrode was fixed at 3.7 mm, the placement and parallelism being ensured with a custom-made laser-cut PMMA support.

The electrodes were connected to the impedance analyzer (MFIA 5 MHz, Zurich instrument) with alligator clips both in the 2- and 4-electrode configurations. Impedance spectra were acquired between 20 Hz and 400 kHz (300 points in total, evenly spaced on a logarithmic scale). An RMS voltage of 250 mV was used to carry out the EIS: this value is low enough to ensure that the tissue response is linear.

Only one sample per configuration was measured.

### Pre/post electroporation measurements—planar electrodes

For the second experiment reported in the section «DRT of potato samples», the samples were similarly cut from potato slices using a round punch (10 mm in diameter) to ensure an identical surface for each. The thickness of the sample was controlled at 5 mm ± 1 mm.

Samples were placed on a sample holder (Solartron analytical, 12962a) at room temperature, sandwiched between two parallel circular brass electrodes. The test bench being equipped with a calliper, the thickness of the samples was precisely measured at this stage, as the distance between the electrodes. This thickness value was used to calculate the voltage to be applied, which is equal to the product of the desired electric field and the height of the sample.

Rapid scans of the impedance spectrum were measured to check the stabilization of the sample. Small variations in impedance were observed during the first few minutes, which can be explained by the pressure exerted on the samples during clamping.

Once the stabilization was achieved, a first spectrum was acquired before electroporation with the same parameters as the preliminary measurement, from 20 Hz to 400 kHz (300 points evenly spaced on a logarithm scale, RMS voltage of 250 mV).

The impedance analyzer was then disconnected from the sample holder to connect the electroporator (Electrocell B15, Leroy Biotech). Electroporation was performed immediately, the voltage being chosen according to the desired electric field value i.e. 60, 100, 200, 300, 400, 500, 750 and 1000 V/cm.

Samples were subjected to a standard electroporation protocol, the European Standard Operating Procedure of Electrochemotherapy (ESOPE), which consists in a series of 8 rectangular pulses with 100 $\mu$s duration separated by 1 s. Chronographs of current and voltage during the procedure were recorded to ensure the proper application of the electric field (see Supplementary Material, part I). It should be noted that ESOPE is not intended for plant electroporation; it is in fact a protocol designed for the treatment of cancerous tumors^18^. However, the potato is used here as a biological model to test the DRT in the context of electroporation; 100 $\mu s$ pulses with an electric field of several hundred V/cm induce electroporation in potatoes^19^.

The impedance analyzer was then connected again, and another spectrum was acquired. The measurements were taken approximately 1 minute after the end of the pulse series. At this point, post-electroporation phenomena are slow (several minutes^20^) compared to the EIS acquisition time (a few seconds). The system can therefore be considered linear time invariant, which is a required condition for performing EIS.

Three samples were tested per electric field level. The final dataset therefore consists in 24 spectra respectively before and after electroporation.

It is worth noting that the DC resistance in Eq. (6), which is used in this study to investigate the effects of electroporation, is not directly derived from the measurements, as these are taken between 20 Hz and 400 kHz. The DC resistance is extrapolated from the mathematical model introduced in the theoretical background section: it refers here to the resistance of the tissue that would be measured in steady state, in the absence of interference from the electrodes.

## Preliminary measurements with 2- and 4-electrode systems

**Fig. 1:**
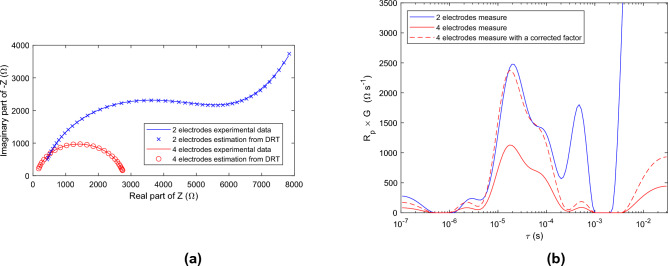
EIS and DRT obtained from measurements performed on a potato sample using 2- and 4-electrode systems, both designed with stainless steel needles. (**a**) Nyquist plot of the experimental impedance spectrum recorded for $N = 300$ frequency points; Nyquist plot of the impedance calculated a posteriori using Eq. (1) is also reported, after the distribution $g(\tau )$ has been estimated by DRT. (b) DRT computed using $M = 120$ step functions in the interval $\tau \in \left[ 10^{-7}\text {s},~10^{-1.5}\text {s}\right]$ and $\lambda = 0.1$ in the penalized regression problem; the displayed distribution is $R_p \times G(log(\tau )) = R_p \times \tau g(\tau )$. The red dotted curve is obtained by applying a correction factor to the DRT obtained with the 4-electrode system in order to compensate for the difference in geometry factor with the 2-electrode system.

DRT is first used to separate the contribution of the electrode/tissue interface from that of the tissue in the spectroscopy data. Two different experiments were performed on native potato samples with 2- and 4-electrode systems. The impedance spectrum was recorded between 20 Hz and 400 kHz and these experimental data were used to compute the DRT (see Fig. 1).

The DRT for the 2-electrode system reveals different relaxation processes at different time scales:

(i) ionic diffusion at the interface tissue/electrode, visible for $\tau > 2~10^{-3}$ s. The ionic diffusion is very slow and cannot be completely caught in the present analysis. Moreover, DRT is not the most suitable tool to capture this phenomenon that is not fundamentally related to a first order relaxation process. It is possible to construct a Distribution of Diffusion Times using a mathematical tool similar to DRT, where first-order relaxation processes are replaced by Warburg elements^21^. However, DRT is sufficient here to identify the time interval specific to diffusion phenomena; a technique described below is then used to eliminate these parasitic effects.
(ii) charge transfer combined with non-faradic effect due to the double layer at the interface tissue/electrode, visible for $2~10^{-4}$ s $< \tau < 10^{-3}$ s.
(iii) dispersions due to the tissue response, visible for $10^{-6}$ s $< \tau < 2~10^{-4}$ s. This part of the DRT is further analyzed in the section «DRT of potato samples».

This interpretation is confirmed when analyzing the results of the 4-electrode system. The 4-electrode device is designed so that current flows between the outer electrodes, while the impedance between the inner electrodes is high to measure voltage. In this way, the polarization of the inner electrodes at the interface with the tissue is eliminated, and the measured impedance reflects only the tissue contribution.

The DRT extracted from the impedance spectrum measured with the 4-electrode system shows that the processes related to the electrode/tissue interface, identified previously for $\tau > 2~10^{-4}$ s, are significantly reduced compared to the 2-electrode system. The peaks due to diffusion and charge transfer/double layer virtually disappear; all that remains is the response of the biological tissue. Note that an additional DRT has been computed by multiplying the DRT of the 4-electrode measurements by a correction factor, set empirically to compensate for the different geometry factors between the 2-electrode and 4-electrode systems. The corrected DRT is close to the DRT obtained with the 2-electrode system for the time interval related to the tissue response, i.e. $10^{-6}$ s $< \tau < 2~10^{-4}$ s. However, the DRT are not perfectly superimposed: there are heterogeneities in the potato and the distribution of the electric field inside the sample is not considered in the same way between the two systems.

Interestingly, the DRT representation enables to separate the contribution of relaxation processes specific to the tissue, from those induced at the electrode/tissue interface when a 2-electrode system is used. According to the Fig. 1b, the limit value $\tau _{max}$ between these two parts of the DRT is around $2~10^{-4}$ s. However, there is a slight overlap between DRT specific to the tissue and that due to the charge transfer/double layer process at the electrode/tissue interface. This latter peak could be suppressed by modeling it with a Gaussian distribution (see the section «Theorerical background»).

**Fig. 2:**
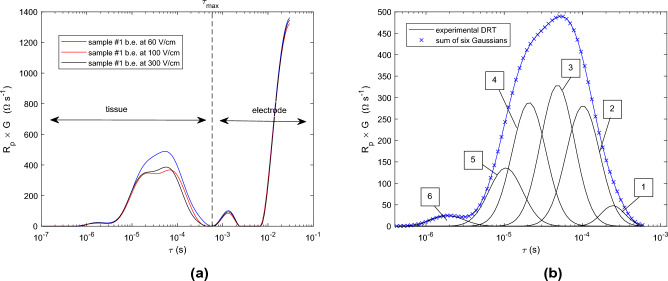
(**a**) DRT computed from the experimental spectrum for the 2-electrode designed with flat brass electrodes, and for 3 different samples of native potato; abbreviation b.e. in the caption is for before electroporation. (**b**) Decomposition with 6 Gaussian distributions for tissue DRT in blue in the figure on the left.

The rest of the study is based on a 2-electrode system consisting of two flat brass electrodes, as described in the section «Materials and methods». The geometry and material of the electrodes alter the electrochemical behavior at the tissue/electrode interface. Figure 2a shows that the electrode polarization processes in the system composed of two flat brass electrodes are slower than in the systems with stainless steel needles. In addition, the part of the DRT related to the tissue can be visually dissociated from that of the electrode/tissue interface, without any overlap: the limit value $\tau _{max}$ between these two parts of the DRT is $6~10^{-4}$ s.

We can also see in Fig. 2a that the DRT is slightly different between samples of the same potato, which again shows that the potato tissue is not homogeneous.

It is worth noting that it is also possible to remove the contribution of the electrodes using traditional EIS. Among the existing methods^22^, the mathematical subtraction method would be the most appropriate for the experimental protocol we have developed. This involves modeling the electrode/tissue interface with an impedance; the model with a resistance in series with a capacitance or the model with a recap are commonly used^23^. However, this method requires setting a priori a model with several parameters to be estimated.

## DRT of potato samples

### DRT analysis before electroporation

In the following, DRT built from measurements made with the 2-electrode system is analyzed focusing on the $\tau$ time interval due to tissue only. We propose to model it by a sum of six Gaussian distributions with respect to the log-normal scale $log(\tau )$ in order to reproduce the complex shape of the tissue DRT. An illustration is displayed in Fig. 2b.

To justify the choice of six Gaussians, F-tests^24^ were performed with an increasing number of Gaussians. The F statistic is given by the formula $\left( RSS_{k-1} - RSS_k\right) /RSS_k \times \left( n-3k - 1\right) /3$, where *n* is the number of time intervals in the DTR and $RSS_k$ the sum of the residual squares between the DTR with the experimental data and the model with *k* Gaussians; there is a factor 3 since there are three parameters (mean, variance and amplitude) for a given Gaussian. The results are $F =$ 39, 168, 66 and 17 for $k =$ 4, 5, 6, and 7, respectively. There is a sharp increase in the F statistic between $k =$ 4 and 5: the difference between the two models is that the peak in the DRT tail (see Fig. 2b) is not captured by a Gaussian for $k =$ 4, unlike the case $k =$ 5. Nevertheless, the F statistic is high for all the values *k*, leading to a p-value close to 0. This would mean that all the Gaussians would be significant in describing the tissue DRT.

However, as mentioned above, at least 5 Gaussians are needed to capture the peak in the DRT tail. Another F-test has been performed focusing on the DRT tail, i.e. for $\tau<$ 6 $10^{-6}$ s (see Fig. 2b). The calculation of the sum of the residual squares is then reduced to a number of time intervals $n_{tail}$ = 26. The results are $F_{tail}$ = 105, 4.8 and 1.7 for $k =$ 5, 6, and 7, respectively; the corresponding p-value is 0, 0.03 and 0.28 for $k =$ 5, 6 and 7, respectively. The p-value is high for $k =$ 7 (> 0.05), which means that adding a seventh Gaussian does not provide any significant information on the DRT tail. For ease of interpretation, the model has therefore been limited to six Gaussians.

#### Qualitative interpretation

Over the frequency range chosen in this study, the literature broadly considers two types of dispersion: $\alpha$ dispersion, mainly due to counterions on the surface of cell membrane, and $\beta$ dispersion, mainly due to cell membrane polarization. However, this classification is somewhat simplistic, as the contents of cells are rich in organelles, organic macromolecules that can undergo polarization in the presence of an external electric field^7,9^.

Identifying precisely the dispersions associated with the six Gaussian distributions is a delicate task, due to the difficulty of conducting control experiments. The following interpretation has been developed based on qualitative considerations reported in the literature, as well as on theoretical elements.

The Gaussian numbered 1 is attributed to the $\alpha$ dispersion. The origin of this dispersion is sometimes confused; the reason is that it is difficult to identify its contribution using traditional spectroscopy impedance^15^. In the approach proposed in this work, effects at the electrode/tissue interface observed when $\tau > 6~10^{-4}$ s have been withdrawn from the DRT study. The first dispersion is then necessarily the $\alpha$ dispersion. The most widely accepted theory for this dispersion involves the lateral diffusion of the counterion atmosphere that surrounds cells^25^. For a spherical single cell, this process basically leads to a first order dielectric dispersion; the time constant for an isolated spherical particle is $r^2/(2ukT)$ where *r* is the radius of the particle or more generally the curvature, *u* the mobility, *k* Boltzmann constant and *T* the temperature. However even for a single cell, there is a distribution of relaxation times because of the variation of electrostatic effects at the membrane surface. Note that the diffusion of counterions at the cell surface behaves differently from the diffusion commonly observed at the electrode/tissue interface, the latter being generally modeled with a Warburg element while the former is equivalent to a RQ element. However, lateral diffusion of counterions is generally not sufficient to fully explain the characteristics of $\alpha$ dispersion in biological tissues^26^; other features, such as the presence of gap junctions, can cause $\alpha$ dispersion^7^.

The Gaussians numbered 2 to 4 are attributed to the $\beta$ dispersion related to the polarization of cells membrane. Adding the 3 Gaussians, the relaxation times spread over more than a decade. The time constant for interfacial polarization of an isolated spherical cell is $r^{cell} C_{m}^{cell} \times (1/\sigma _{c}^{cell} + 1/2\sigma _0)$ where $r^{cell}$ is the cell radius, $\sigma _{c}^{cell}$ (respectively $\sigma _0$) the intracellular (respectively bulk medium) conductivity and $C_{m}^{cell}$ the membrane capacitance (see Supplementary Material, part III). The membrane capacitance is broadly the same between cells, the membrane being composed of a lipid bilayer about 5 nm thick. Cells size, shape and orientation can be more variable: in the case of potato, the average size is around 100 $\mu$m with polygonal shapes. This variability partly explains the spread of the $\beta$ dispersion.

This model of the $\beta$ dispersion neglects the presence of the double layer responsible for the $\alpha$ dispersion described above. At the time scale corresponding to the Gaussians numbered 2 to 4, the lateral diffusion of the counterions does not have time to occur. Instead, the double layer acts as a $C_{dl}^{cell}$ capacitance that is added in series with the membrane capacitance (see Supplementary Material, parts II and III). The total capacitance for a single cell is therefore $C_{m}^{cell} /(1 + C_{m}^{cell}/C_{dl}^{cell})$. $C_{dl}^{cell}$ value is in the range of $C_m^{cell}$ value and even larger^27^: the double layer modifies the relaxation time for the interfacial polarization. In addition, the $C_{dl}^{cell}$ capacity may fluctuate according to the electrostatic state of membrane surface. These fluctuations can also explain the variability of time constants for the $\beta$ dispersion.

**Fig. 3:**
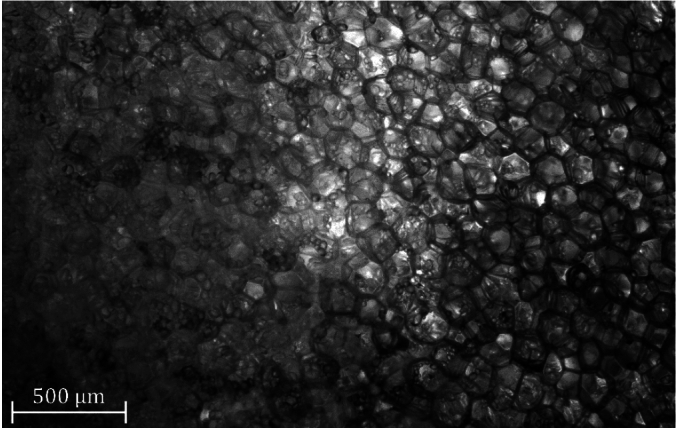
Potato slice that covers the internal phloem (left part of the image) and the pitch (right part of the image). Cells in the pitch contains a few starch granules while cells in the phloem contains large amount of starch.

Another explanation for the spread of the $\beta$ dispersion is the content of the cells: the polarization of proteins and other organic macromolecules may contribute to $\beta$ dispersion^9^. Potato cells store more or less starch depending on their location in the potato^28^. Figure 3 shows a microscopic section where the starch content is highly variable between cells. Starch granules are composed of polysaccharide macromolecules (linear-chain amylose and branched-chain amylopectin). They have a lenticular shape and can reach a size of 100 $\upmu$m^29^. The intracellular medium can therefore have different electrical properties, depending on the size and number of starch granules.

Moreover, the surface of starch granules is charged, resulting in the presence of a double layer, as evidenced by potential zeta measurements reported in the literature ^30^. This double layer introduces another dispersion related to the Gaussian numbered 5. The relaxation time for the counterion dispersion of starch granules would be very short compared to the one for the cells: it is a function of the square of the particle curvature and the granules are smaller and rounder than the cells. An interfacial polarization could also be possible: the double layer can act as a capacitance $C_{dl}^{starch}$ leading to a dispersion with a time constant that notably depends on the granule radius and the potato starch conductivity.

To the best of our knowledge, the only low-frequency spectroscopic study of potato starch granules has been proposed by Beh et al.^29^. The analysis was carried out with a potato starch suspension: the authors observed dispersion and attributed it to interfacial polarization. This conclusion is questionable as the possibility of a counterion diffusion is not mentioned. As the starch conductivity is expected to be smaller than that of the cytoplasm, the current density would flow tangentially around the starch granules, promoting the lateral diffusion at the surface of the granules.

Because of the double layer, starch granules are isolated for longer time constant than the characteristic time of Gaussian numbered 5. In that case, the current inside the cytoplasm can only flow outside the starch granules. Back to $\beta$ dispersion of cells, it implies that the resistance of the cytoplasm is greater when starch granules are present. The time constant of the interfacial polarization is therefore larger for cells containing starch. By way of illustration, the time constant for cell membrane polarization can be multiplied by a factor of 5 when starch occupies 75$\%$ of the intracellular medium, as shown in the calculation presented in the Supplementary Material, part III. The Gaussian numbered 2 corresponds to cells containing a larger quantity of starch granules than the Gaussians numbered 3 and 4.

The Gaussian numbered 6 is related to a much shorter time constant: the related relaxation process appears at a very small spatial scale. We assume that it is due to the dispersion of cell nuclei. This is consistent with the literature, which indicates that the tail of $\beta$ dispersion can be attributed to cellular organelles^7^.

#### Quantitative analysis

The $R_{pk}$ resistance defined in Eq. 6 was estimated for 6 Gaussians and for 24 samples before electroporation.

First, the heterogeneity of the samples is addressed. The mean value of DC resistance, prior to electroporation and before rescaling, is 928 $\Omega$ for the 24 samples, with a standard deviation of 245 $\Omega$. This variability is partly due to geometric tolerance in the production of samples. This is particularly true for sample thickness, which was measured with an average value of 4.9 mm and a standard deviation of 0.7 mm. The cross-section was much more precise, as the samples were made using a cylindrical metal cutter. The heterogeneity of the tissue composition is also an issue, even though we took samples from the center of the potatoes.

**Fig. 4:**
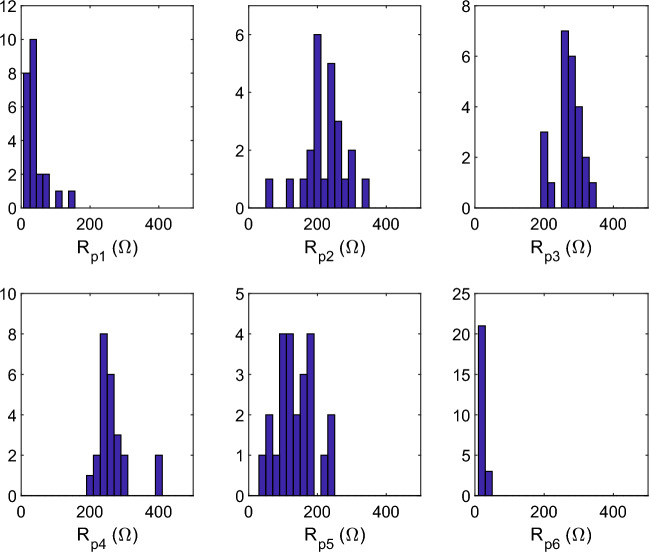
$R_{pk}$ values deduced from the DRT of 24 potato samples before electroporation.

To weight variability between samples, the results were rescaled with respect to a reference DC resistance $R_{ref} = 1000~\Omega$. The impedance measurements were multiplied by a factor $R_{ref}/R_0$ where $R_0$ is the DC resistance measured before correction. Fig. 4 shows the results and Table 1 reports the statistics.

$R_{p6}$ has small mean values. This is because the related relaxation processes take place in a very small volume compared to the other processes. The Gaussian numbered 6 concerns cell nuclei, which are 10 to 100 times smaller than the cells. In addition, the nuclear membrane is leaky to ions, resulting in small polarization.

$R_{p1}$, to a lesser extent than $R_{p6}$, has also small values. The Gaussian numbered 1 is associated to the $\alpha$ dispersion which occurs on an extremely thin layer of a few nanometers on the cell membrane surface (see Supplementary Material, part II).

$R_{p5}$ is highly disparate compared to $R_{p2}$, $R_{p3}$ or $R_{p4}$ when computing the ratio standard deviation/mean. The Gaussian numbered 5 is related to the dispersion of starch granules, which show great variability in terms of number and size. The Gaussians numbered 2, 3 and 4 are related to the $\beta$ dispersion of cells in the presence of a various amount of starch: the variability of the starch content is therefore distributed over the three Gaussians.

**Table 1:** Statistics for $R_{pk}$ in Fig. 4.

	$R_{p1}$	$R_{p2}$	$R_{p3}$	$R_{p4}$	$R_{p5}$	$R_{p6}$
Mean ($\Omega$)	39	221	270	266	139	26
Standard deviation ($\Omega$)	34	59	37	49	54	4

### DRT analysis after electroporation

Samples were subjected to a series of 8 rectangular pulses with 100 $\mu$s duration separated by 1 s (ESOPE).

#### Reversible and irreversible electroporation

**Fig. 5:**
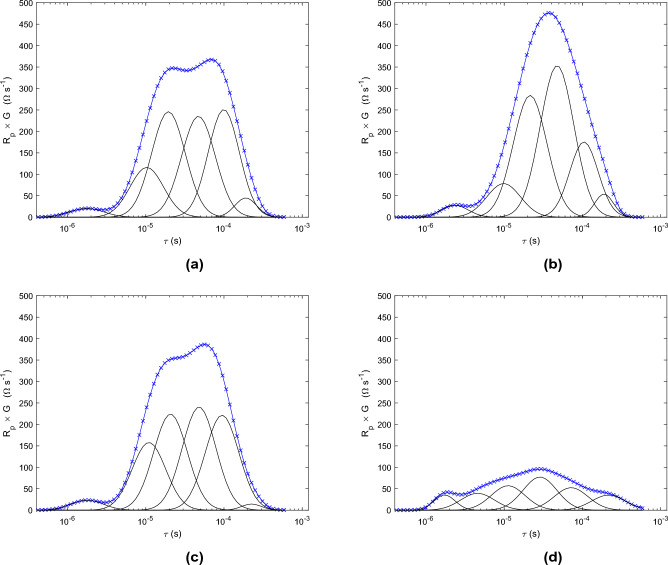
(**a**) DRT before and (**b**) after electroporation at 100 V/cm; DC resistance is 1.13 k$\Omega$ before electroporation and 1.16 k$\Omega$ after electroporation. (**c**) DRT before and (**d**) after electroporation at 300 V/cm; DC resistance is 1.12 k$\Omega$ before electroporation and 0.39 k$\Omega$ after electroporation.

Figure 5b shows an illustration of the DRT obtained at 100 V/cm. Gaussians numbered 2 to 4 are significantly modified compared to the DRT before electroporation, as shown in Fig. 5a . At the same time, DC resistance remains unchanged after electroporation. These are the characteristics of reversible electroporation, as shown by equation SI6 in the discussion on reversible electroporation in the Supplementary Material, part II. It should be noted that the tissue model used to derive equation SI6 is similar to the one used to model electroporation ^31^. However, a capacitance has been added to describe the double layer at the surface of the cell membrane; this has an influence because the DRT is derived from spectroscopy measurements performed with signals of low amplitude compared to the pulses applied during electroporation. The membrane regains its insulating properties once the electric field has been switched off. However, the TMP evolves over a long period during which ion channels are activated to restore the resting potential^20^. Under these conditions, the double layer capacitance $C_{dl}^{cell}$ changes over time. The time constant of the cell membrane polarization is then modified, which explains the difference observed on Gaussians numbered 2, 3 and 4.

Gaussian numbered 1 is also modified since the counterion cloud evolves on the membrane surface after the electric field has been turned off.

Gaussian numbered 5 is also changed: we assume that a small part of this distribution is related to the $\beta$ dispersion of cells. This assumption is consistent with the analysis of DRT as a function of electric field proposed in the following paragraph.

Gaussian numbered 6 is almost unchanged, showing that the cell nuclei are not affected.

Figure 5d shows an illustration of the DRT obtained at 300 V/cm. The entire DRT has been modified after electroporation. In addition, the DC resistance has decreased by a factor of 2.9. These are the characteristics of irreversible electroporation (see Eq. SI7 and the discussion on irreversible electroporation in the Supplementary Material, part II). A large number of pores remain after the pulse has been turned off, and the membrane permanently loses its insulating properties. The double layer on the starch granules is also affected. Under these conditions, the Gaussians numbered from 2 to 5 decrease sharply in amplitude.

Gaussian numbered 1 is still present, proving that there is still a counterion atmosphere around the cells.

Finally, Gaussian numbered 6 is the less modified: electroporation has reached almost no cell nuclei.

#### Response with respect to the electric field

Different electric field amplitudes were applied: 60, 100, 200, 300, 400, 500, 750 and 1000 V/cm. For each electric field value, the experiment was repeated on three samples.

In order to compare the different samples, the impedance spectra obtained before and after electroporation have been rescaled according to the DC resistance obtained before electroporation and the reference value $R_{ref} =1000~\Omega$. For instance, let be $R_0^{0V/cm}$ the DC resistance before electroporation for a sample used in an experiment at 100 V/cm; impedance spectra recorded before and after electroporation are multiplied by $R_{ref}/R_0^{0V/cm}$ for rescaling.

**Fig. 6:**
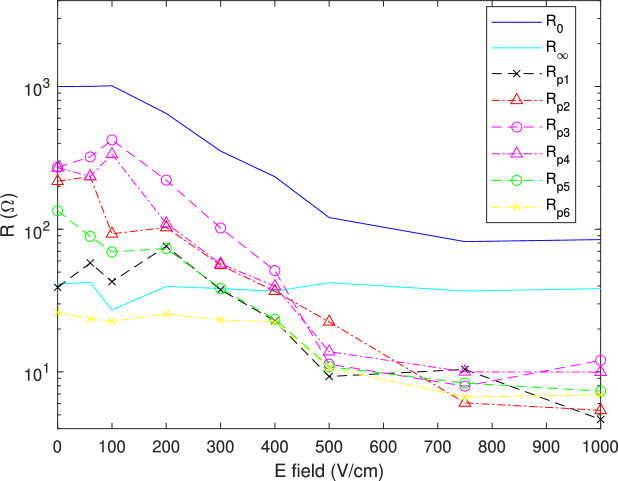
Evolution of the resistances $R_{pk}$ for different amplitudes of the electric field.

Figure 6 shows the evolution of $R_{pk}$ after electroporation for the different electric field values. The reversible electroporation threshold is around 100 V/cm. Under this threshold, the DC resistance remains virtually constant. However, there are some changes in the distribution of Gaussians.

Between 0 and 60 V/cm, there is a small drop of $R_{p4}$ and $R_{p5}$; at the same time, there is a small increase of $R_{p2}$ and $R_{p3}$. Reversible electroporation modifies membrane polarization, as evidenced by the evolution of TMP several minutes after the pulse is turned off^20^. As a consequence, the value of the double-layer capacitance $C_{dl}^{cell}$ also varies, resulting in a change in the time constants related to the interfacial polarization of cell membrane (see Eq. SI6). At the moment the impedance spectrum was acquired at 60 V/cm, Gaussian numbered 4 had lightly decreased in favor of Gaussians numbered 2 and 3. This means that the time constants of the $\beta$ dispersion have increased overall. Gaussian numbered 5 has also decreased. However, this Gaussian is a priori related to the dispersion of the starch granules. The most likely hypothesis is that a small part of this distribution concerns $\beta$ dispersion of cell membrane. As the time constant of this dispersion tends to increase overall, the characteristic time of the few $\beta$ dispersions that were captured by Gaussian numbered 5 are shifted to Gaussian numbered 4.

Between 60 and 100 V/cm, the Gaussian numbered 2 is dramatically reduced while the Gaussian numbered 3 and 4 increase. This means that, at the moment the impedance spectrum was acquired at 100 V/cm, the time constants of the cell membrane polarization have decreased overall. This conclusion is the opposite of the one drawn for an electric field of 60 V/cm. However, it is not contradictory. The evolution of the TMP after the pulse is turned off is not monotonic: Blazic et al observe that the TMP may transiently be below the resting potential (depolarization) and then later exceed this value (hyperpolarization)^20^. Ionic concentrations in the vicinity of the membrane can therefore decrease or increase compared to the physiological equilibrium value, which can decrease or increase the value of the double layer capacitance.

For an electric field value of 100 V/cm, Gaussians numbered 3 and 4 are the main contribution to DC resistance. Gaussian numbered 3 is related to cells with a larger content of starch than Gaussian numbered 4 (see Supplementary Material, part III).

Irreversible electroporation begins to occur at an electric field amplitude higher than 100 V/cm. We observe that $R_{p4}$ decreases faster than $R_{p3}$. This is consistent with the theory of electroporation: in the presence of starch granules, the time constant of the interfacial polarization is larger; this makes the cell membrane less sensitive to the electric field, leading to a higher electroporation threshold (see Supplementary Material, part IV).

$R_{p1}$ starts decreasing for a larger electric field around 300 V/cm; $R_{p5}$ decreases in the same way. This tends to show that the electrical charge, due to the adsorption of ions on the surface of the cell membranes and granules, is dramatically altered for an electric field higher than 200 V/cm.

$R_{p6}$ starts decreasing for an even larger electric field around 500 V/cm, which can be explained by the very small size of the nuclei.

It is worth noting that there is no substantial change in $R_\infty$ according to the electric field, meaning that the conductivity of the different compartments is overall not modified during electroporation. This result is in accordance with equations SI6 and SI7 for reversible and irreversible electroporation.

The study has been conducted without taking thermal effects into account, as these are very limited. The electrical pulses cause the temperature of the samples to rise due to the Joule effect. The energy absorbed is given by the formula $U I \Delta t$, where *U* is the voltage, *I* the current, and $\Delta t$ the cumulative duration of the pulses. The larger rise in temperature is expected at 1000 V/cm; the chronograms are given in the Supplementary Material part I for one of the three samples studied, which had a thickness of 4.5 mm and was subjected to pulses of 450 V. In this experiment, the current increases slightly with the number of pulses, from 10.5 A to 12 A. Setting $U =$ 450 V, $I =$ 12 A, and $\Delta t$ = 8$\times$100 $\mu$s = 0.8 ms, the absorbed energy is 4.3 J, which is slightly overestimated. Furthermore, the thermal capacity of the sample can be calculated using the formula $\rho c_p V_{sample}$, where $\rho$ is the mass density of potatoes, $c_p$ the thermal capacity, and $V_{sample}$ the volume of the sample. Setting $\rho ~=$ 1062 $kg/m^3$ and $c_p =$ 3.77 kJ kg$^{-1}$ K$^{-1}$^32^, the thermal capacity of the system is 1.4 J/K for a sample with a height of 4.5 mm and a diameter of 10 mm. The temperature rise is then 3.1 $^{\circ }$C for pulses at 1000 V/cm, under adiabatic conditions. For pulses at 500 V/cm, the temperature increase is only 0.7 $^{\circ }$C.

The real temperature is even lower due to heat transfer, mainly through the brass electrodes. The diffusion time is given by the formula $l^2\rho c_p/k$, where *l* is the characteristic length of the system and *k* the thermal conductivity. Setting $k =$ 0.55 W m$^{-1}$ K$^{-1}$^32^, the diffusion time is 37 s for a characteristic length equal to half the sample height. The sample has time to cool down a little since EIS is performed 1 minute after the end of the pulses.

The values of the conductivity temperature coefficient $\alpha$ vary between 0.025/K and 0.03/K for intact and completely damaged potato tissue, respectively^33^. For the experiment at 500 V/cm (respectively 1000 V/cm), there would be an increase of less than 2 $\%$ (respectively 9 $\%$) in conductivity due to temperature, while DC resistance decreases by a factor of 8 (respectively 12) compared to the value before electroporation, as shown in Fig. 6.

## Conclusion

In this article, a DRT analysis was carried out on potato samples subjected to electroporation. Firstly, the low kinetics associated with electrode polarization at the tissue interface make it possible to separate its contribution from that of the tissue in a 2-electrode system, using a time constant threshold. This technique may prove more interesting than those used when working with EIS data in the frequency domain.

Secondly, the DRT of untreated potato samples reveals four dispersions: the $\alpha$ dispersion due to counterions on the membrane surface, the $\beta$ dispersion due to polarization of cell membranes, a dispersion due to starch granules contained in the cells and a dispersion due to cell nuclei. The $\beta$ dispersion due to the cell membrane polarization is spread over three Gaussians. This is not only due to the variability in cell size and shape: the content in starch granules varies between cells, which significantly modifies the electrical properties of the cytoplasm.

Thirdly, DRT can be used to better understand which tissue compartments are affected as a function of electric field amplitude. The potato sample is subjected to reversible electroporation for an electric field lower than 100 V/cm. There is no change in DC resistance: the membrane regains its electric properties once the voltage pulse is turned off. However, the electrostatic equilibrium of the cell membrane is altered by the high electric field, which mainly modifies the double layer on the membrane surface. This changes the DRT related to $\alpha$ dispersion. The $\beta$ dispersion is also modified: the double layer acts as a capacitance and it affects the relaxation time of the membrane polarization.

For an electric field higher than 100 V/cm, where irreversible electroporation occurs, DRT analysis shows that the threshold is different depending on the content of starch granules in cells, which is confirmed by electroporation simulations. This partly explains why the DC resistance gradually decreases by a factor of 8 between 100 and 500 V/cm. In addition, the $\alpha$ dispersion and the dispersion related to starch granules, which are due to ionic charges adsorbed on the particle surface, tend to disappear for an electric field around 300 V/cm. Finally, the dispersion related to cell nuclei is altered for an electric field around 500 V/cm.

One of the prospects is to use DRT to analyze impedance data in vivo. For example in the case of liver electroporation, decomposition using Gaussian distributions would enable a better distinction to be made between the effects on liver cells, tumor cells and blood vessels. The features extracted by DRT would also enable better use of machine learning tools by feeding them with relevant data.

## Supplementary Information

Supplementary Information.

## Data Availability

The datasets generated and analysed during the current study are available from the corresponding author on reasonable request.
