# Supplementary material for: Distribution of relaxation times as a tool to monitor tissue electroporation
Source: Sci Rep. 2025 Nov 24;15:41678. doi: 10.1038/s41598-025-25647-4 (PMC12644780; doi:10.1038/s41598-025-25647-4)
Supplement: Supplementary file 1 — Supplementary Information. [file 41598_2025_25647_MOESM1_ESM.pdf]

Supplementary Material, part I: voltage/current chronographs

This section presents some chronographs recorded during the application of ESOPE to potato samples.

The tests were carried out with an Electrocell B15 device (Leroy Biotech) that provides voltage and current measurements. ESOPE consists in 8 pulses of 100 μs with a repetition period of 1 s.

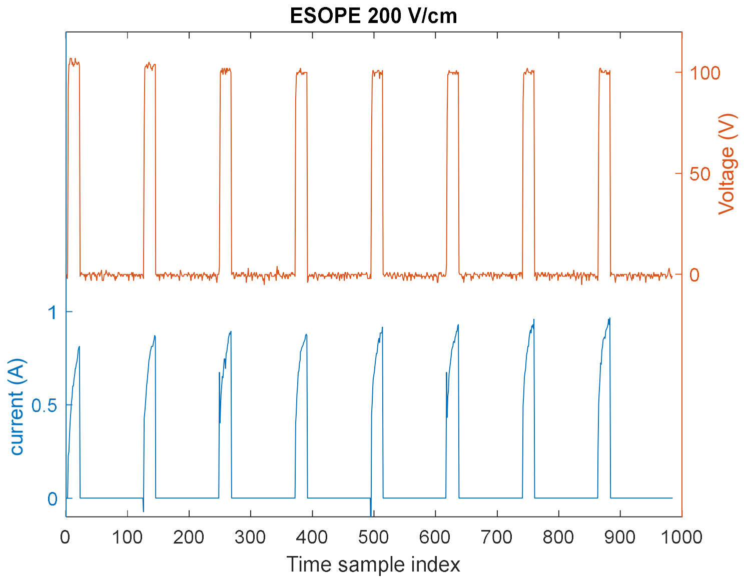

SI Figure 1: voltage and current recorded during the application of ESOPE for 200 V/cm; the thickness of the potato sample was 5 mm. The abscissa is given according to the time sample number; for a given pulse, 21 time samples are recorded at 5 μs intervals during the application of voltage (100 μs), followed by 101 samples recorded at 9.995 ms intervals when the voltage is cut off (approximately 1 s).

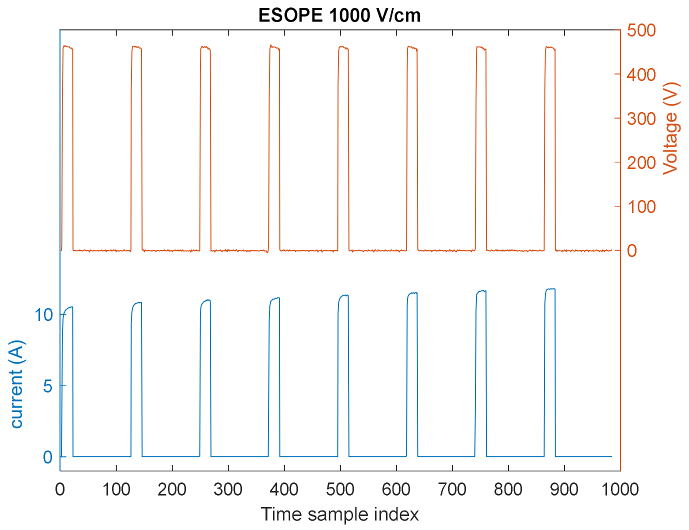

SI Figure 2: voltage and current recorded during the application of ESOPE for 1000 V/cm; the thickness of the potato sample was 4.5 mm. The abscissa is given according to the time sample number, as in SI Fig. 1.

Supplementary Material, part II: DRT for interfacial polarization of cell membrane

A simplified tissue model is proposed to understand the changes caused by electroporation. This model describes a single process for the cell membrane polarization, assuming that all cells are identical. It is not intended to reproduce the exact response of the tissue.

Equivalent circuit

The equivalent circuit in SI Fig. 3 is suitable for spectroscopic characterization of the interfacial polarization of cell membrane at the tissue scale. There are similarities with the equivalent circuit used to model electroporation of a tissue subjected to a high electric field [31].

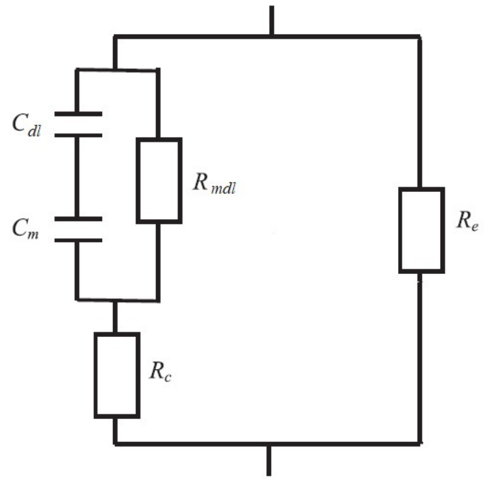

SI Figure 3: Equivalent circuit of the tissue for spectroscopy measurement in the range of frequency related to interfacial polarization of cell membrane

The current density within the tissue is divided into extracellular and intracellular currents. The extracellular medium is a purely conductive medium modeled by the resistance R_e. The intracellular current density flows through the cells. Cells are made up of an intracellular medium, modeled by the resistance R_c, surrounded by a membrane. The membrane is insulating under physiological conditions, which is modeled by the capacitance C_m.

The introduction of a phase constant element instead of the capacitance would be more appropriate to describe the distribution of relaxation times related to the β dispersion. Here, the capacitance model is preferred as it provides a qualitative interpretation of the change in the time constant after electroporation

Capacitance due to the double layer

Compared to the equivalent circuit that can be used to model electroporation [31], the effect of the counterions cloud due to the ions adsorbed on the membrane surface is added by means of the capacitance C_{dl}. The counterions are distributed in a double layer: in the first layer counterions are immobile, facing the adsorbed ions on the membrane surface; in the second layer, counterions are mobile and spread over a distance equal to the Debye length according to the Poisson-Boltzmann equation[1]. At low frequencies, the cloud of mobile counterions induces an α dispersion, reflecting a lateral diffusion phenomenon. At higher frequencies, counterions have no time to diffuse. Instead, the counterion cloud produces a capacitive effect.

There are two contributions to the C_{dl} capacitance. Firstly, the immobile counterion layer behaves like a conventional planar capacitor. Secondly, the mobile counterion layer behaves like a second capacitor in series; however, the small signal injected for the spectroscopy measurement acts as a small perturbation around the equilibrium of the mobile charge distribution. In the static case, the total charge σ_d of the mobile counterions depends on the surface potential ψ_d (which is close to the well-known ζ potential). For a symmetric electrolyte of valence z, the solution of Boltzmann-Poisson equation gives:

$$\sigma_d = \frac{2V_B \kappa \varepsilon}{z} sinh\left(\frac{z\psi_d}{2V_B}\right) \qquad \text{(SI1)}$$

where ε is the permittivity, $V_B = k_B T/e$ = 25 mV at 25°C and $1/\kappa$ is the Debye length. For a symmetric electrolyte of valence z, Debye length is given by:

$$1/\kappa = \sqrt{\frac{V_B \varepsilon}{F z^2 c}} \qquad \text{(SI2)}$$

where F is the Faraday constant and c the bulk ionic concentration.

Equation SI1 is valid when the Debye length is very small compared to the cell radius, which is the case for biological cells[2] where the Debye length is of the order of 1 nm. When small perturbation occurs as in spectroscopy measurement, the part of the double layer capacitance due to the presence of the mobile counterions is provided by the dynamic capacitance $\partial\sigma_d/\partial\psi_d$. Experiments carried out in physiological conditions[3] give values for ζ potential of no more than 40 mV; the potential Ψ_d is therefore not greater than 40 mV. Assuming the equilibrium value ψ_d < 50 mV leads to:

$$\frac{\partial\sigma_d}{\partial\psi_d} \approx \kappa\varepsilon \qquad \text{(SI3)}$$

[1] D'Orlye, F. Ph.D. thesis, Pierre Marie Curie University (2009).
[2] Wennerstrom, H., Estrada, E. V., Danielsson, J. and Oliveberg, M. Colloidal stability of the living cell. Proc. Natl. Acad. Sci. U.S.A. 117, 10113-10121 (2020).
[3] Hughes, M. The cellular zeta potential: cell electrophysiology beyond the membrane. Integr. Biol. 16 (2024).

After a high electric field is applied, the large interfacial polarization of the membrane may change the distribution of adsorbed ions on the membrane surface. This may significantly alter the potential ψ_d. in addition to changing the TMP. Moreover, the ionic concentration in the diffuse double layer changes, modifying the Debye length. Under these conditions, the double layer capacity C_{dl} is expected to change after electroporation.

Resistance due to the creation of pores

When the electric field exceeds a threshold, pores form. After the pulse has stopped, pores with a radius of around 20 nm may persist[4]. Current can flow through these pores, making the membrane more conductive. Moreover, there is no cloud of counterions on the (fictitious) surface of the pores: ions can then move with the same mobility as in the bulk medium. The resistance R_{mdl} models these two effects

DRT for reversible and irreversible electroporation

The analysis of the equivalent circuit in SI Fig. 3 differs depending on whether electroporation is reversible or irreversible.

Reversible electroporation

In this case, few pores may remain opened after the pulse is switched off. The membrane properties are the same as before the pulse was applied: the membrane remains highly resistive, $R_{mdl} \to +\infty$

The equivalent impedance can then be expressed as follows:

$$Z(\omega) = \frac{R_e R_c}{R_e + R_c} + \frac{{R_e}^2}{R_e + R_c} \times \frac{1}{1 + j\omega(R_e + R_c)C_m/(1 + C_m/C_{dl})} \tag{SI4}$$

The DRT of Equation SI4 gives a single process of first order:

$$Z(\omega) = R_\infty + R_p \frac{1}{1 + j\omega\tau} \tag{SI5}$$

with

[4] Krassowska, W. & Filev, P. Modeling electroporation in a single cell. Biophys. J. 92, 404-417 (2007).

$$R_\infty = \frac{R_e R_c}{R_e + R_c}$$
$$R_p = \frac{{R_e}^2}{R_e + R_c} \qquad \text{(SI6)}$$
$$\tau = (R_e + R_c) \times \frac{C_m}{1 + C_m/C_{dl}}$$

In the case of reversible electroporation, the TMP may vary for several minutes after the pulse is switched off in order to restore the physiological conditions [20]. In the absence of an external electric field, the resting transmembrane potential depends on ionic concentrations close to the membrane. As the TMP varies after electroporation, so do ionic concentrations. As a result, the distribution of the counterion cloud is altered.

From a spectroscopic point of view, a change is observed in the α dispersion, but also in the β dispersion. For the latter, only the time constant τ changes with the value of C_{dl} since the capacitance depends on the ionic concentration at the vicinity of the membranes. However, there is no change in R_c and R_e, as the double layer involves only a small volume compared to the intra and extra-cellular domains; R_p in the DRT is unchanged.

R_∞ is not modified. It gives the equivalent resistance under an infinite frequency: in that situation, the current flows through the double layer and the membrane.

Ultimately, the DC resistance given by $R_\infty + R_p$ is unchanged with reversible electroporation.

Irreversible electroporation

In this case, pores persist in the membrane: R_{mdl} takes a finite value; C_{dl} is also changed in a large extend compared to the reversible electroporation.

The impedance for the equivalent circuit is still related to the DRT of a single process of first order given Equation SI5, with different parameters:

$$R_\infty = \frac{R_e R_c}{R_e + R_c}$$
$$R_p = \frac{{R_e}^2}{R_e + R_c} \times \frac{1}{1 + (R_e + R_c)/R_{mdl}} \qquad \text{(SI7)}$$
$$\tau = \frac{R_e + R_c}{1 + (R_e + R_c)/R_{mdl}} \times \frac{C_m}{1 + C_m/C_{dl}}$$

R_{mdl} decreases with the increasing effects of irreversible electroporation, so does the value of R_p.

As in the reversible case, R_∞ is not modified. Ultimately, the DC resistance given by $R_\infty + R_p$ decreases as irreversible electroporation progresses.

The time constant τ also decreases with R_{mdl}. But this may be compensated for by the capacitance C_{dl}, which may increase at the same time.

Supplementary Material, part III: Response of a single cell according to the amount of starch

In this section, the response of an isolated cell subjected to a variable uniform electric field of low amplitude is calculated from Maxwell's equations as a function of starch content. It is shown that the relaxation time of the interfacial polarization depends on the starch volume.

The solution for a single cell (without starch granule) is provided in the transient regime in [31]. First, reconsider the problem in the harmonic regime.

Take a spherical cell of radius r^{cell} immerged in a bulk medium of conductivity σ_0. The intracellular conductivity is σ_c^{cell} and a membrane of capacitance C_m^{cell} (in F/m²) surrounds the cell.

The problem to solve reads:

$$\begin{aligned} &\Delta\phi = 0 \quad r \neq r^{cell} \\ &[\phi]_{r=r^{cell}} = v_m{}^{cell} = \frac{1}{jC_m{}^{cell}\omega} J_{r^{cell}} \\ &J_{r^{cell}} = \sigma_c{}^{cell} \left.\frac{\partial\phi}{\partial r}\right|_{r-r^{cell}=0^-} = \sigma_0 \left.\frac{\partial\phi}{\partial r}\right|_{r-r^{cell}=0^+} \end{aligned} \qquad \text{(SI8)}$$

The cell is excited by a uniform external field E_0. Then, $\phi = \phi_{ext} + \phi_{ind}$ with $\phi_{ext} = -E_0\, r\cos(\theta)$ and $\phi_{ind}(r) \to 0$ when $r \to +\infty$

Because of the invariance according to the angle variable θ, the general solution for the potential is given by:

$$\phi = \left(A\,r + \frac{B}{r^2}\right)cos(\theta) \qquad \text{(SI9)}$$

Coefficient A and B are different for the cytoplasm and for the bulk medium. These coefficients can be expressed from boundary conditions. Finally, the TMP reads:

$$v_m{}^{cell} = \frac{{}^{3}/_{2}\,E_0 r^{cell}}{1 + j\tau^{cell}\omega} cos(\theta) \qquad \text{(SI10)}$$

with the time constant

$$\tau^{cell} = \left(R_c{}^{cell} + R_0{}^{cell}\right) \times C_m{}^{cell}$$

where $R_c^{cell} = r^{cell}/\sigma_c^{cell}$ and $R_0^{cell} = r^{cell}/2\sigma_0$ are the resistances (in Ω m²) of the cytoplasm and the bulk medium.

As explained in the section "DRT of potato samples," starch granules are surrounded by a double layer. This acts as an insulating effect in the frequency range where the interfacial polarization of the cell membrane is studied.

Assume an isolated starch granule of radius r^{stach} is added inside the cytoplasm. The problem to solve reads:

$$\begin{aligned}
&\Delta\phi = 0 \quad r \neq r^{cell}, r > r^{starch} \\
&[\phi]_{r=r^{cell}} = v_m{}^{cell} = \frac{1}{jC_m{}^{cell}\omega} J_{r^{cell}} \\
&J_{r^{cell}} = \sigma_c{}^{cell} \frac{\partial\phi}{\partial r}\bigg|_{r-r^{cell}=0^-} = \sigma_0 \frac{\partial\phi}{\partial r}\bigg|_{r-r^{cell}=0^+} \\
&\sigma_c{}^{cell} \frac{\partial\phi}{\partial r}\bigg|_{r=r^{starch}} = 0
\end{aligned} \quad \text{(SI11)}$$

The TMP is expressed similarly as in Equation (SI10) except the time constant is

$$\tau^{cell+starch} = \left(R_c{}^{cell} \frac{2+x}{2-2x} + R_0{}^{cell} \frac{2-x}{2-2x}\right) \times C_m{}^{cell} \quad \text{(SI12)}$$

with

$$x = \left(\frac{r^{starch}}{r^{cell}}\right)^3$$

The resistance of the cytoplasm, and to a lesser extent that of the bulk medium, necessarily increases in the presence of starch granules. The time constant for the interfacial polarization of the cell membrane is therefore necessarily greater in the presence of starch. It increases dramatically when x tends towards 1, i.e. when the starch content becomes preponderant.

When for example the granule occupies half the volume of the cytoplasm (x = 0.5), $R_c{}^{cell}$ increases by a factor 2.5 and $R_0{}^{cell}$ by a factor 1.5. Under these conditions, the time constant of the interfacial polarization increases by a factor between 1.5 and 2.5.

When the granule occupies 75% of the cytoplasm volume (x = 0.75), the time constant of the interfacial polarization increases by a factor between 2.5 and 5.5.

The theoretical results were obtained without taking into account the double layer that may be present on the surface of the cell membrane. This behaves like a $C_{dl}{}^{cell}$ capacitance. When this capacitance is taken into account, the previous expressions can be rewritten by changing the term $C_m{}^{cell}$ to $C_m{}^{cell}/(1+ C_m{}^{cell}/ C_{dl}{}^{cell})$.

Supplementary Material, part IV: Effect of the amount of starch on the electroporation rate

In this section, an electroporation calculation is performed at tissue scale, modulating the properties of the intracellular medium according to the result of part III. It is shown that, for a given pulse amplitude, electroporation is more advanced in the case where the cells contain the least starch.

During electroporation, the electric field induced between the electrodes by the high-voltage pulse is very high compared with the electrostatic field that maintains the double layer under physiological conditions. Under these conditions, there is no effect of the double layer. Therefore the model used for electroporation at tissue scale is close to that presented in part II, except the capacitance C_{dl} no longer appears; the capacitance C_m is in parallel with the resistance R_{mdl} (that we note here as R_m for convenience). This is the model proposed in [31].

As the system consists of two flat electrodes facing each other, the electric field induced between the electrodes is uniform when the sample is considered to be homogeneous. The problem can be solved using the voltage v, which is directly linked to the electric field E by the relationship E = v/l, where l is the distance between the electrodes. Similarly, the current i is linked to the current density J by the relationship J = i/S where S is the cross-sectional area of the electrodes.

Setting v is the total voltage across the equivalent circuit of the tissue and $2u_m$ the voltage across the set C_m parallel to R_m, the electrical equations are as follows:

$$C_m \frac{du_m}{dt} + \left(\frac{1}{R_c} + \frac{1}{R_m}\right) u_m = \frac{v}{2R_c} \qquad \text{(SI13)}$$

Assuming $C_m = \varepsilon_m S/l$, $R_c = l/\sigma_c S$ and $R_m = l/\sigma_m S$, the result is:

$$\varepsilon_m \frac{du_m}{dt} + (\sigma_c + \sigma_m) u_m = \frac{\sigma_c}{2} v \qquad \text{(SI14)}$$

ε_m (respectively σ_m) is the equivalent permittivity (respectively conductivity) of the membrane at the tissue scale. σ_c is the equivalent conductivity of the cytoplasm at the tissue scale. Note that these properties are different from the ones defined at the cell scale because of renormalization [31]. Typical values are reported in SI Table 1.

Renormalization can also be used to extract the homogenized TMP $v_m = 2\, r^{cell}/l \times u_m$. In addition, the membrane conductivity depends on the homogenized TMP. Using the model of pores creation as in [31], one has:

$$\sigma_m = \sigma_{m0} + \sigma_{m1} g(v_m) X(v_m, t) \qquad \text{(SI15)}$$

where

$$\frac{dX}{dt} = \frac{\beta(v_m) - X}{\tau(v_m)}$$

$$\beta(v_m) = \frac{N_0}{N_{max}}\left(e^{q\left(\frac{v_m}{v_{ep}}\right)^2} - 1\right)$$

$$\tau(v_m) = \frac{N_0}{\alpha} e^{(q-1)\left(\frac{v_m}{v_{ep}}\right)^2}$$

and

$$g(v_m) = \frac{e^{\frac{F}{RT}v_m} + 1}{\frac{w_0 e^{\left(w_0 - n\frac{F}{RT}v_m\right)} w_0 - n\frac{F}{RT}v_m}{w_0 - n\frac{F}{RT}v_m} e^{\frac{F}{RT}v_m} - \frac{w_0 e^{\left(w_0 + n\frac{F}{RT}v_m\right)} w_0 + n\frac{F}{RT}v_m}{w_0 + n\frac{F}{RT}v_m}}$$

Parameters are defined in SI Table 1.

SI Table 1: parameters for the simulation of electroporation at the tissue scale [31].

Quantity	Symbol	Value
Equivalent intracellular medium conductivity at the tissue scale	σ_c	0.17 or 0.35 S/m
Equivalent membrane permittivity at the tissue scale	ε_m	60000 × ε_0
Equivalent membrane conductivity at the tissue scale before electroporation	σ_{m0}	0.01 S/m
Equivalent membrane conductivity at the tissue scale for the formation of pores	σ_{m1}	6.0 S/m
Barrier energy within the pore for the electrodiffusion term	w_0	0.85
Relative entrance length of pores for the electrodiffusion term	n	0.15
Initial density of pores when v_m = 0 *Volt*	N_0	1.5 × 10^5 cm^{-2}
Maximum density of pores	N_{max}	10^{10} cm^{-2}
Characteristic voltage for electroporation	v_{ep}	0.11 Volt
Parameter in the function τ (v_m)	α	10^5 cm^{-2} s^{-1}
Parameter in the function β (v_m)	q	2.5

X is a measure of pore creation: X = 0 when no pore is created and X = 1 when the maximum number N_{max} of pores is reached.

The dynamics of X is modeled by a differential equation of first order, where in particular the time constant and the final value depend on the TMP.

Membrane polarization is a process that depends on the properties of the cytoplasm. In the presence of starch granules, the characteristic time to establish TMP before electroporation is longer, as shown by the formulae derived in part III. In that case, it is expected that the electroporation phenomenon will then be less marked.

To test this hypothesis, two simulations were carried out with the parameters reported in SI Table 1. Only the conductivity of the cytoplasm changes between the two simulations: it is 0.17 S/m and 0.35 S/m respectively; it is twice as low in the first case to simulate the presence of starch granules.

SI Figure 4 shows that the TMP takes longer to reach the critical value[5] $4 \times V_{ep}$ = 0.44 Volts when the conductivity of the cytoplasm is lower: 1.06 μs when σ_c = 0.17 S/m against 0.53 μs when σ_c = 0.35 S/m. As a result, the electroporation rate X is lower when the conductivity of the cytoplasm is lower: 0.13 when σ_c = 0.17 S/m against 0.25 when σ_c = 0.35 S/m.

For the same value of electric field, the effect of electroporation will be more marked for cells containing the least starch.

[5] K.A. DeBruin, W. Krassowska, Modeling Electroporation in a Single Cell. I. Effects of Field Strength and Rest Potential, Biophysical Journal 77 (1999) 1213–1224.

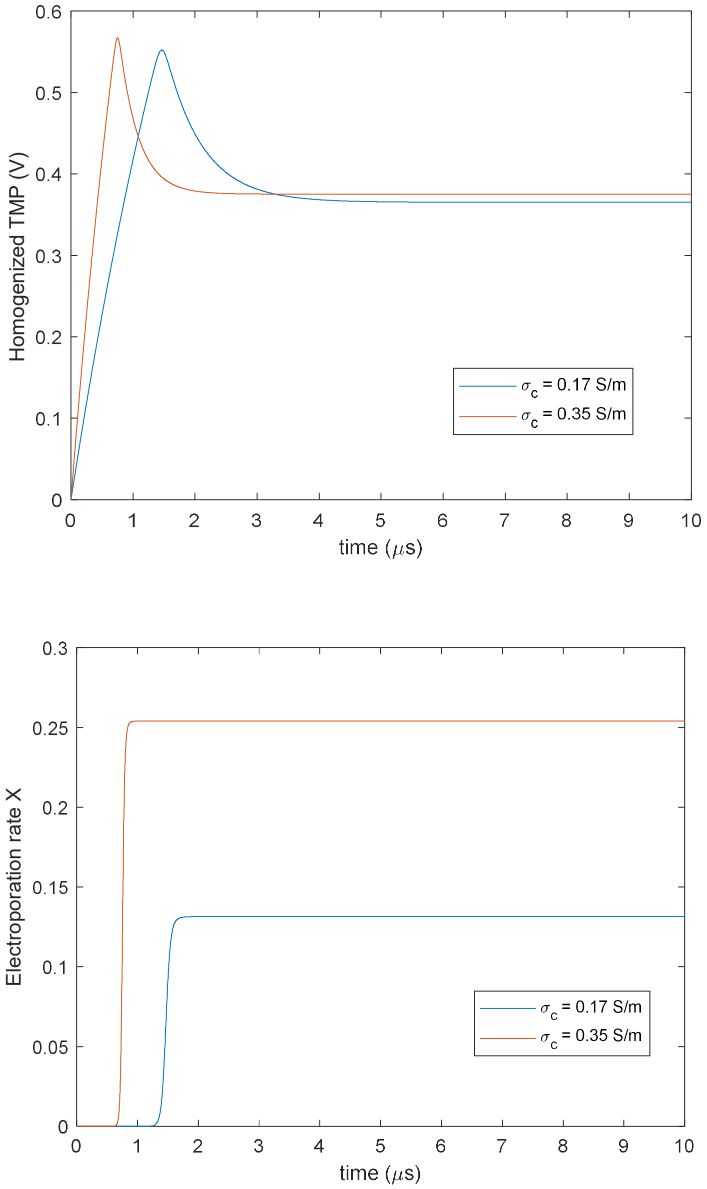

SI Figure 4: Homogenized TMP (on the top) and electroporation rate (on the bottom) for an electric field of 300 V/cm and a cell radius of 50 μm. The smaller value for the cytoplasm conductivity models the presence of starch granules. Other parameters are reported in SI Table 1.
